# AN INNOVATIVE MEDICAL LEGAL PARTNERSHIP TO SERVE COMMUNITY-DWELLING OLDER ADULTS

**DOI:** 10.1093/geroni/igac059.847

**Published:** 2022-12-20

**Authors:** Rachel Lessem, Tamara Kushnir Groman

**Affiliations:** CJE SeniorLife, Chicago, Illinois, United States; CJE SeniorLife, Chicago, Illinois, United States

## Abstract

According to the Legal Services Corporation, each year, 56% of older adults have at least one civil legal problem yet 87% receive inadequate or no professional legal help for their civil legal problems. Unmet legal needs can have cascading catastrophic effects on the health and well-being of older adults. To address this, we took the innovative approach of creating a Medical-Legal Partnership (MLP) within a community based social service agency on aging. MLPs have been successful by addressing the social determinates of health. However, previous studies have not investigated MLPs aimed at older adults. We used a single arm, pretest-posttest design to investigate the effects and feasibility of providing legal services to community dwelling older adults. The MLP served over 88 clients referred both internally from the agency as well as external partners. The participants had a mean age of 73, (age range 45-98, n=88), were 60% female. African Americans represent 9% of the clients, 7% are Hispanic/Latino and 74% Caucasian. Additionally, 5% were veterans. 52% of clients were at or below the Federal Poverty line at baseline. 50% of clients reported greater financial stability as a result of legal services. 75% reported improved health of between 1 and 3 points on a 5 point likert scale (pre-test=2.75; post-test 3.9 p<.01). This pilot project demonstrates a successful intervention to work across professional silos coordinating lawyers, medical providers and social workers to better meet the legal needs of older adults by creating MLPs in social service agencies to support their well-being.

